# Fundamental Aspects of Skin Cancer Drugs via Degree-Based Chemical Bonding Topological Descriptors

**DOI:** 10.3390/molecules28093684

**Published:** 2023-04-24

**Authors:** Abdul Rauf Khan, Nadeem ul Hassan Awan, Muhammad Usman Ghani, Sayed M. Eldin, Hanen Karamti, Ahmed H. Jawhari, Yousef E. Mukhrish

**Affiliations:** 1 Department of Mathematics, Faculty of Science, Ghazi University, Dera Ghazi Khan 32200, Pakistan; 2 Institute of Mathematics, Khawaja Fareed University of Engineering & Information Technology, Abu Dhabi Road, Rahim Yar Khan 64200, Pakistan; 3 Faculty of Engineering and Technology, Future University in Egypt, New Cairo 11835, Egypt; 4 Department of Computer Sciences, College of Computer and Information Sciences, Princess Nourah bint Abdulrahman University, P.O. Box 84428, Riyadh 11671, Saudi Arabia; 5 Department of Chemistry, Faculty of Science, Jazan University, P.O. Box 45142, Jazan 45142, Saudi Arabia

**Keywords:** skin cancer, chemical bonding, drugs, QSPR model, calculations

## Abstract

Due to significant advancements being made in the field of drug design, the use of topological descriptors remains the primary approach. When combined with QSPR models, descriptors illustrate a molecule’s chemical properties numerically. Numbers relating to chemical composition topological indices are structures that link chemical composition to physical characteristics. This research concentrates on the analysis of curvilinear regression models and degree-based topological descriptors for thirteen skin cancer drugs. The physicochemical characteristics of the skin cancer drugs are examined while regression models are built for computed index values. An analysis is performed for several significant results based on the acquired data.

## 1. Introduction

**Figure 1:**
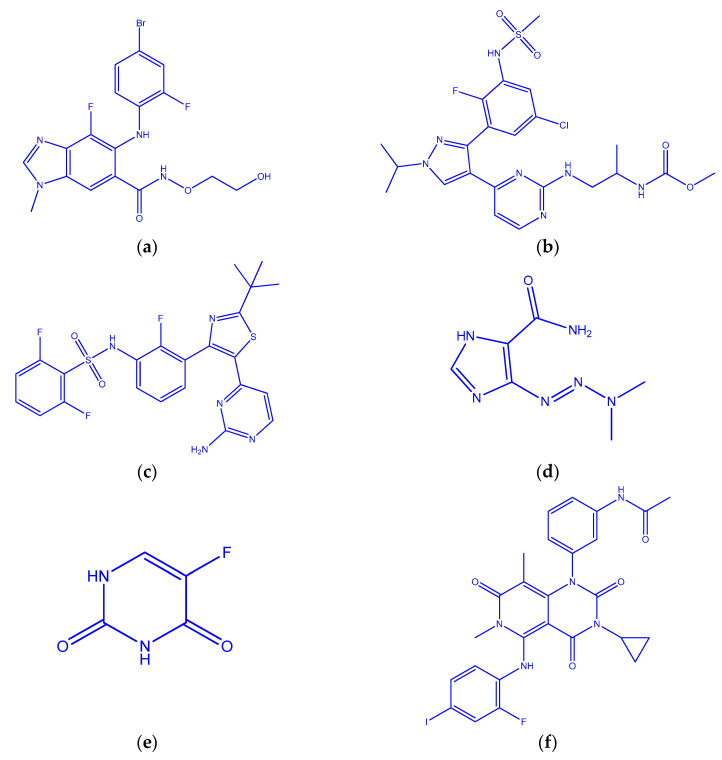

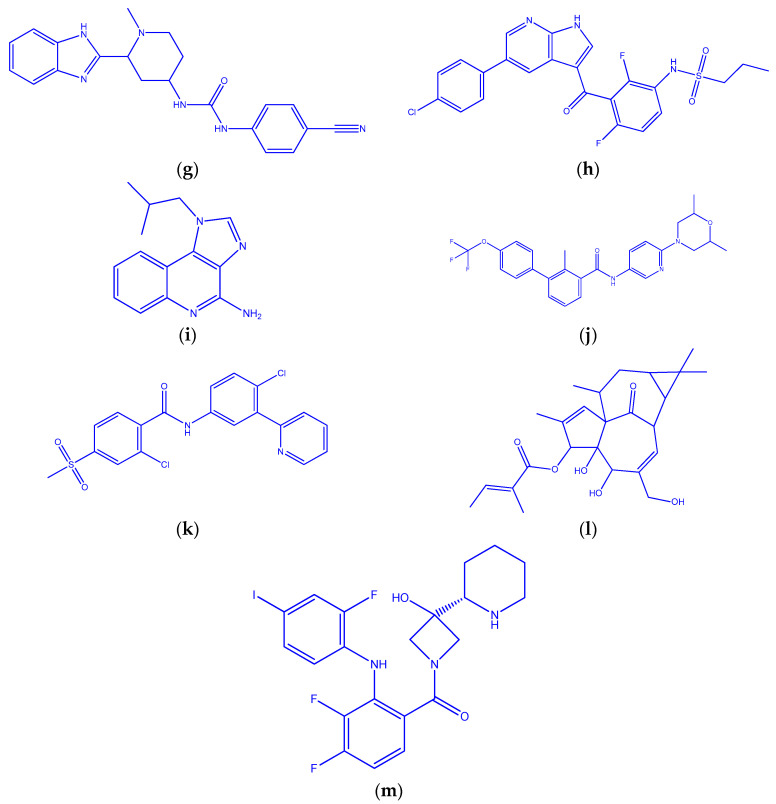
Molecular structures. (**a**) Binimetinib. (**b**) Encorafenib. (**c**) Dabrafenib. (**d**) Dacarbazine. (**e**) Fluorouracil. (**f**) Trametinib. (**g**) Daurismo. *(***h**) Vemurafenib. (**i**) Imiquimod. (**j**) Odomzo. (**k**) Vismodegib. (**l**) Picato. (**m**) Cobimetinib.

The largest organ in the body is the skin. It offers protection from heat, sunburn, harm, and illness. Additionally, it regulates the temperature of our body and water, and stores vitamin D and fat. Three different types of cells make up the epidermis. The majority of the epidermis is made up of thin, flat cells called squamous cells. Melanin, the pigment that imparts skin its natural color, is produced by them. Melanocytes produce higher pigment whenever skin is exposed to the sun, which darkens or tans the skin. Blood and lymphatic vessels, hair follicles, and glands are all found in the dermis. Nonmelanoma skin malignancies also include squamous cell carcinoma and basal cell carcinoma. Skin cancer can develop anywhere around the body, although it is most frequent on the face, neck, hands, and arms because of exposure to sunlight. The most prevalent cancer in the country, skin cancer has a high morbidity and fatality rate [1]. A chronic autoimmune disorder called inflammatory bowel disease (IBD) is linked to a higher risk of developing skin cancer. This study aims to evaluate skin cancer knowledge and risk factors in IBD patients. We will also evaluate how the patients are currently protecting their skin. Through this study, we hope to uncover any knowledge gaps in the patient population with inflammatory bowel disease in regard to how to prevent skin cancer. Skin cancer seems to be developing more frequently each year in terms of new instances. Usually, these skin tumors are curable. Since at least 40 years ago, there has been an increase in the number of new melanoma cases. Melanoma might be more difficult to treat and is more prone to spread to neighboring tissues and other body parts. The risk factors for melanoma and malignancies differ. It is unknown whether avoiding the sun, applying sunscreen, or donning protective clothing when outdoors reduces the risk of skin cancer. Basal cell carcinoma (BCC) and squamous cell carcinoma are two types of nonmelanoma skin cancer that are prevalent around the world (SCC). Every year, there are roughly two to three million NMSCs worldwide. BCC is the most prevalent cancer in the United States (U.S.) [2]. According to estimates, there were two million new NMSC cases in the U.S. in 2012, and treatment delays resulted in severe morbidity [3]. Significant morbidity can also result from postponing the treatment of NMSCs. Fair skin color and blond hair have a high propensity for sunburn and are all extrinsic and intrinsic risk factors for the development of skin cancer [4]. The sickness also had a significant impact on a global scale. New pharmaceuticals are developed and studied by scientists, and their findings are a difficult undertaking because it is an expensive, time-consuming, and challenging discipline. To treat and stop this fatal condition, numerous drug studies are mandated, and numerous drug tests are carried out to combat these fatal diseases [5]. It necessitates prompt discovery and the swift use of medication in order to beat the disease. Thirteen medicines binimetinib, encorafenib, dabrafenib, dacarbazine, fluorouracil, trametinib, daurismo, vemurafenib, imiquimod, odomzo, vismodegib, picato, and cobimetinib are the most beneficial drugs for a community’s well-being. Figure 1 depicts the structure of the above-mentioned drugs. 

A kinase inhibitor called dabrafenib is used to treat people with some forms of thyroid cancer, non-small cell lung cancer, and melanoma. The same mutation was also applied to metastatic non-small cell lung cancer. It was used in conjunction with trametinib as a primary or adjuvant treatment for unresectable or metastatic melanoma. The combination of dabrafenib and trametinib is also recommended for the treatment of locally progressed or metastatic anaplastic thyroid cancer in addition to melanoma. Dabrafenib and trametinib each inhibit a distinct pathway effector, which boosts response rates and reduces resistance without causing cumulative damage. Dabrafenib with trametinib is the first combination therapy for thyroid cancer that has been shown to have substantial clinical action in BRAF V600E-mutated anaplastic thyroid carcinoma and is well tolerated. These results signify a significant therapeutic advance for this rare condition. Malignant melanoma and Hodgkin’s disease are both treated with the antineoplastic drug dacarbazine. An anti-cancer substance, it has significant anti-melanoma effects. For the treatment of malignant melanoma, clinical trials are being conducted with dacarbazine and oblimersen. When dacarbazine is administered intravenously, the volume of distribution surpasses the water content of the entire body, indicating localization in bodily tissue, most likely the liver. In 6 hours, on average 40% of the administered dose of unaltered DTIC is excreted cumulatively in urine. Dacarbazine is not significantly bound to human plasma protein at therapeutic dosages. A pyrimidine analogue known as fluorouracil is injected into the body to treat cancer and treat basal cell carcinomas, a pyrimidine analogue that functions as an antimetabolite against cancer. By preventing the conversion of deoxyuridylic acid to thymidylic acid by the enzyme thymidylate synthetase, it prevents the creation of DNA. Antineoplastic anti-metabolite fluorouracil impersonates purines or pyrimidines, which are used to make DNA. They prohibit these chemicals from incorporating into DNA during the cell cycle’s “S” phase, which halts healthy cell growth and division. An FDA-approved kinase inhibitor called trametinib is used to treat individuals with some forms of thyroid cancer, non-small cell lung cancer, and melanoma. Kinase inhibitor trametinib dimethyl sulfoxide is used. The thyroid’s tissues can develop cancerous cells as a result of thyroid cancer. An uncommon and deadly kind of thyroid cancer is anaplastic thyroid cancer. According to the National Institutes of Health (NIH), there will be 53,990 new instances of thyroid cancer in 2018 and 2060 fatalities from the condition. Trametinib is an anti-cancer drug that suppresses cell growth and causes apoptosis, or “programmed cell death”, both of which are crucial for the treatment of cancer. A sonic hedgehog receptor inhibitor called a glasdegib is used to treat newly diagnosed acute myeloid leukemia in those over 75 who are unable to undergo intensive chemotherapy. This series of chemicals’ high lipophilicity sparked interest in further modification. According to this investigation, the presence of p-cyano ureas had favorable physicochemical and pharmacokinetic characteristics that led to the development of glasdegib. In xenograft models used in preclinical investigations, glasdegib significantly decreased the burden of leukemic stem cells and the cell population expressing leukemic stem cell markers. In the same study, 31% of the patients with acute myeloid leukemia and 8% of those with acute myeloid leukemia experienced stable disease states. The most recent clinical trial demonstrated that glasdegib produced an overall survival of 8.3 months, which was nearly twice as long as what had been seen in patients receiving low-dose cytarabine therapy. Moreover, reports of dose-dependent QTc prolongation in patients receiving glasdegib have been made. By attaching to the mutant BRAF’s ATP-binding domain, it performs its task. Moreover, 3 vemurafenib was co-developed by Roche and Plexxikon and on 17 August 2011 it received FDA approval under the name Hoffmann La Roche. Upon licensure, Roche and Genentech started a significant development program. According to all available reports, vemurafenib almost entirely inhibits the MAPK pathway warts inside the vagina, penis, or rectum that cannot be treated. Actinic keratoses is a skin disorder that affects the face and scalp that is also treated with imiquimod. Imiquimod is also effective in treating some skin cancers known as superficial basal cell carcinomas. Imiquimod is especially helpful for places like the face and lower legs where surgery or other therapies would be difficult, complicated, or otherwise unfavorable. The antineoplastic drug sonidegib is used to treat locally advanced recurring basal cell carcinoma (BCC) after surgery and radiation therapy or in situations when these treatments are contraindicated. The smoothened antagonism-based hedgehog signaling pathway inhibitor called sonidegib was created by Novartis as a cancer treatment. The FDA gave it the go-ahead to treat basal cell cancer. Sonidegib has been demonstrated to inhibit SMO, a transmembrane protein involved in the transmission of the Hh signal. In some animal models, this led to the suppression of Hh signaling as well as anti-tumor action. Sonidegib-treated mice in a transgenic mouse model of islet cell neoplasms had 95% less tumor volume than untreated mice. On 30 January 2012, vismodegib was approved by the FDA for the treatment of adult basal cell carcinoma and suppresses the hedgehog signaling system. To block the hedgehog signaling pathway, vismodegib specifically binds to and inhibits the transmembrane protein smoothened homolog (SMO). Actinic keratosis is treated with the topical medication ingenol mebutate. However, it is uncertain how ingenol mebutate works pharmacologically to cause cell death in actinic keratosis.

Kanwal et al. in [6] examined the behaviors of some drug structures, such as those within anti-cancer drugs, using multi-criteria decision-making methodologies like TOPSIS and SAW. This study is the first to use specific MCDM algorithms to rate various medication architectures. A ranking technique called TOPSIS analyses decision-making issues both quantitatively and qualitatively. Several novel topological descriptors for cerium oxide were computed by Zaman S. et al. [7]. Since they may be chemically coupled with other carbon-based materials and through a variety of different elements to create solid covalent connections, carbon nanosheets have a wide range of applications. For the two carbon nanosheets, Asad Ullah et al. [8] computed various novel neighborhood versions of molecular descriptors and derived formulas. Their calculated results show a correlation between the acentric factor and entropy, which makes them effective in QSPR and QSAR analysis with significant accuracy. They are undoubtedly beneficial in comprehending the topology of the understudied nanosheets. In addition, they are significantly better in isomer discrimination than other degree-based indices. The numerical outcomes for the modeling of the boiling point in benzenoid hydrocarbons were reviewed by Ali et al. in their study [9]. These findings demonstrate that the correlation of the first Zagreb eccentricity index’s boiling point in benzenoid hydrocarbons yields better results than the correlation of the second Zagreb eccentricity index. In the class of all connected n-node bipartite networks, they were able to determine the minimal transmission. The parameters are highly helpful in modifying or altering a specific network’s course. As Wang et al. [10] discussed, there has been a threat to creating cancer therapy. Each year, this sickness affects up to 10 million people worldwide. Anti-cancer drugs are those prescribed to patients with cancer, a malignant disease. Many studies illustrate the strong correlation between the boiling, melting, and enthalpy properties of alkanes and the chemical structure of anti-cancer medicines. This study looks at a few antiviral drugs that are regarded to have the potential for treating cancer.

The estimate of some physicochemical properties of these drugs is that TDs are also used in the development of the QSPR models. They carried out the QSPR research, which makes use of curve fitting, and revealed a substantial link with the properties of anti-cancer drugs. Zaman et al. in [11] investigated the physical and chemical parameters of the understudy nanosheet that were examined numerically using the given formulas. The topology of the understudied nanosheet may surely be understood using the results of our computations. These computed indices are completely accurate in QSPR and QSAR analysis since they better reflect an association with the acentric factor and entropy.

The molecular graph represents a molecular structure made up of a collection of endpoints known as atoms or vertices V (G), which are connected by a collection of bonds known as edges E (G). The size and order are the total number of atoms or vertices and the total number of bonds or edges, respectively, in a molecular graph [12]. Typically, to solve various chemical graphs, graph theory and chemistry are combined. Topological descriptors are widely used in the fields of chemical graph theory and mathematical chemistry and also have significant use in QSPR analysis.

Parveen et al. [13] applied the QSPR model to predict the physical properties of diabetes disease drugs. Colakoglu [14] discusses earlier studies on possible medications for the treatment of COVID-19. This technique works best for predicting discoveries because it is an expensive and complicated phenomenon. The blood cancer medication results of Nasir et al. in [15] show by QSPR modeling a strong correlation between the characteristics of drugs and TDs. Parveen et al. in [16] examined the chemical components that make up RA medicines using targeted analyses and meticulously designed topological indexes. Numerous investigations have discovered a clear connection between the molecular structures of chemical compounds and medications and their chemical properties, such as their boiling and melting points. The modeling of cardiovascular drugs is thoroughly studied with the aid of topological descriptors in [17]. Autoimmune disease vitiligo drugs are discussed in [18].

The above studies inspired us to work on the current study challenge by using different topological indices for different chemical structures and, in the present study, we premeditated degree-based topological descriptors on skin cancer drugs.

We used the following topological descriptors (TDs):

**Definition** **1.** *ABC index* [19] *G is given under:*

(1) $$ABC\left(G\right)=\underset{uv\in E\left(G\right)}{\sum }\sqrt{\frac{d_{u+}d_{v}-2}{d_{u}d_{v}}}$$

**Definition** **2.** *Randic index* [20] *is given under:*

(2) $$RA\left(G\right)=\underset{uv\in E\left(G\right)}{\sum }\sqrt{\frac{1}{d_{u}d_{v}}}$$

**Definition** **3.** *Sum connectivity index* [21] *is given under:*

(3) $$S\left(G\right)=\underset{uv\in E\left(G\right)}{\sum }\sqrt{\frac{1}{d_{u}+d_{v}}}$$

**Definition** **4.** *GA index* [22] *is given under:*

(4) $$GA\left(G\right)=\underset{uv\in E\left(G\right)}{\sum }\frac{2\sqrt{d_{u}d_{v}}}{d_{u}+d_{v}}$$

**Definition** **5.** *Zagreb indices* [23] *are given under:*

(5) $$M_{1}\left(G\right)=\underset{uv\in E\left(G\right)}{\sum }\left(d_{u}+d_{v}\right)$$

(6) $$M_{2}\left(G\right)=\underset{uv\in E\left(G\right)}{\sum }\left(d_{u}d_{v}\right)$$

**Definition** **6.** *Harmonic index* [24] *of G is given under:*

(7) $$H\left(G\right)=\underset{uv\in E\left(G\right)}{\sum }\frac{2}{d_{u}+d_{v}}$$

**Definition** **7.** *Hyper Zagreb index* [25] *is given under:*

(8) $$HM\left(G\right)=\underset{uv\in E\left(G\right)}{\sum }{\left(d_{u}+d_{v}\right)}^{2}$$

**Definition** **8.** *Forgotten index* [26] *is given under:*

(9) $$F\left(G\right)=\underset{uv\in E\left(G\right)}{\sum }\left[{\left(d_{u}\right)}^{2}+{\left(d_{v}\right)}^{2}\right]$$

We were encouraged to work on the existing research topic by studies on COVID-19, anti-cancer, blood cancer, and the QSPR of different topological descriptors for different drugs. The goal of this project is to investigate how topological descriptors might be used to model drug regimens for treating skin diseases and determine their physical qualities.

## 2. Results and Discussion

In this section, topological descriptors of skin cancer drugs are calculated.

### 2.1. Topological Descriptors of Binimeinib

A drug called binimetinib is used to treat metastatic melanoma with certain mutations. As a result, the growth of tumor cells may be inhibited. A kinase inhibitor called encorafenib is administered to treat metastatic or incurable melanoma with certain mutations [27,28]. The most often found cancer-causing mutation in this gene is the V600E mutant, which has also been discovered in a number of other malignancies, such as non-Hodgkin lymphoma, colorectal cancer, thyroid carcinoma, non-small cell lung carcinoma, hairy cell leukemia, and lung adenocarcinoma. The effectiveness of encorafenib in treating metastatic melanoma has improved.

Using the data from the edge partition, we computed topological descriptors for the binimeinib (BM) in this section [29]. Let the graph BM with edge set *E* and $E_{m,n}$ are edges in $G_{1}\;$ with, $\left|E_{1,2}\right|=1,\;\left|E_{1,3}\;\right|=5,$ $\left|E_{2,2}\;\right|=5,\;\left|E_{2,3}\;\right|=11,\;\left|E_{3,3}\;\right|=7$ [30]. By applying Definitions 1–8 we obtained the results and TDs are given as follows:

$$\mathrm{ABC}(\mathrm{BM})=1\sqrt{\frac{1+2-2}{1\times 2}\;}+5\sqrt{\frac{1+3-2}{1\times 3}\;}+5\sqrt{\frac{2+2-2}{2\times 2}\;}+11\sqrt{\frac{2+3-2}{2\times 3}\;}+7\sqrt{\frac{3+3-2}{3\times 3}\;}=20.77$$

$$RA\left(\mathrm{BM}\right)=1\sqrt{\frac{1}{1\times 2}\;}+5\sqrt{\frac{1}{1\times 3}\;}\;+5\sqrt{\frac{1}{2\times 2}\;}+11\sqrt{\frac{1}{2\times 3}}\;+7\sqrt{\frac{1}{3\times 3}\;}=12.92$$

$$S\left(\mathrm{BM}\right)=\;1\sqrt{\frac{1}{1+2}\;}+5\sqrt{\frac{1}{1+3}\;}\;+5\sqrt{\frac{1}{2+2}\;}+11\sqrt{\frac{1}{2+3}\;}\;\;+7\sqrt{\frac{1}{3+3}\;}=13.35$$

$$GA\left(\mathrm{BM}\right)=\;\frac{2\sqrt{1\times 2}}{1+2}+\frac{10\sqrt{1\times 3}}{1+3}+\frac{10\sqrt{2\times 2}}{2+2}+\frac{22\sqrt{2\times 3}}{2+3}+\frac{14\sqrt{3\times 3}}{3+3}=28.05$$

$$M1\left(\mathrm{BM}\right)=1\left(1+2\right)+5\left(1+3\right)+5\left(2+2\right)+11\left(2+3\right)+7\left(3+3\right)=140$$

$$M2\left(\mathrm{BM}\right)=1\left(1\times 2\right)+5\left(1\times 3\right)+5\left(2\times 2\right)+11\left(2\times 3\right)+7\left(3\times 3\right)=166$$

$$H\left(\mathrm{BM}\right)=1\left(\frac{1}{1+2}\right)+5\left(\frac{1}{1+3}\right)+5\left(\frac{1}{2+2}\right)+11\left(\frac{1}{2+3}\right)+7\left(\;\frac{1}{3+3}\right)=12.40$$

$$HM\left(\mathrm{BM}\right)=1{\left(1+2\right)}^{2}+5{\left(1+3\right)}^{2}+5{\left(2+2\right)}^{2}+11{\left(2+3\right)}^{2}+7{\left(3+3\right)}^{2}=696$$

$$F\left(\mathrm{BM}\right)=1\left(1+4\right)+5\left(1+9\right)+5\left(4+9\right)+11\left(4+9\right)+7\left(9+9\right)=364$$

### 2.2. Topological Descriptors of Encorafenib

Using the data from the edge partition, we computed topological descriptors for the encorafenib (EB) in this section. Let graph of EB with edge set *E*. Let $E_{m,n}$ are edges of $G_{2}$ with $\left|E_{1,2}\;\right|=1\;,\;\left|E_{1,3}\;\right|=6\;,\left|E_{1,4}\;\right|=3,\;\left|E_{2,2}\;\right|=3,\;\left|E_{2,3}\;\right|=18\;,\;\left|E_{2,4}\;\right|=1,\left|E_{3,3}\right|=6\;.$ By applying Definitions 1–8, we obtained the results and TDs are given as follows:

$$\mathrm{ABC}(\mathrm{EB})=1\sqrt{\frac{1+2-2}{1\times 2}\;}+6\sqrt{\frac{1+3-2}{1\times 3}\;}+3\sqrt{\frac{1+4-2}{1\times 4}\;}+3\sqrt{\frac{2+2-2}{2\times 2}\;}+18\sqrt{\frac{2+3-2}{2\times 3}\;}+1\sqrt{\frac{2+4-2}{2\times 4}\;}+6\sqrt{\frac{3+3-2}{3\times 3}\;}=27.76$$

$$RA\left(\mathrm{EB}\right)=1\sqrt{\frac{1}{1\times 2}\;}+6\sqrt{\frac{1}{1\times 3}\;}\;+3\sqrt{\frac{1}{1\times 4}\;}+3\sqrt{\frac{1}{2\times 2}\;}+18\sqrt{\frac{1}{2\times 3}}+1\sqrt{\frac{1}{2\times 4}\;}\;+6\sqrt{\frac{1}{3\times 3}\;}=16.87$$

$$S\left(\mathrm{EB}\right)=1\;\;\sqrt{\frac{1}{1+2}\;}+6\sqrt{\frac{1}{1+3}\;}\;+3\sqrt{\frac{1}{1+4}\;}+3\sqrt{\frac{1}{2+2}\;}+18\sqrt{\frac{1}{2+3}\;}+1\sqrt{\frac{1}{2+4}\;}\;+6\sqrt{\frac{1}{3+3}\;}=17.33$$

$$GA\left(\mathrm{EB}\right)=\;\frac{2\sqrt{1\times 2}}{1+2}+\frac{12\sqrt{1\times 3}}{1+3}+\frac{6\sqrt{1\times 4}}{1+4}+\frac{6\sqrt{2\times 2}}{2+2}+\frac{2\sqrt{2\times 3}}{2+3}+\frac{12\sqrt{2\times 4}}{2+4}+\frac{12\sqrt{3\times 3}}{3+3}=36.12$$

(10) $$M1\left(\mathrm{EB}\right)=1\left(1+2\right)+6\left(1+3\right)+3\left(1+4\right)+3\left(2+2\right)+18\left(2+3\right)+1\left(2+4\right)+6\left(3+3\right)=186$$

$$M2\left(\mathrm{EB}\right)=1\left(1\times 2\right)+6\left(1\times 3\right)+3\left(1\times 4\right)+3\left(2\times 2\right)+18\left(2\times 3\right)+1\left(2\times 4\right)+6\left(3\times 3\right)=214$$

$$H\left(\mathrm{EB}\right)=1\left(\frac{1}{1+2}\right)+6\left(\frac{1}{1+3}\right)+3\left(\frac{1}{1+4}\right)+3\left(\frac{1}{2+2}\right)+18\left(\frac{1}{2+3}\right)+1\left(\frac{1}{2+4}\right)+6\left(\;\frac{1}{3+3}\right)=15.9$$

$$HM\left(\mathrm{EB}\right)=1{\left(1+2\right)}^{2}+6{\left(1+3\right)}^{2}+3{\left(1+4\right)}^{2}++3{\left(2+2\right)}^{2}+18{\left(2+3\right)}^{2}+1{\left(2+4\right)}^{2}+6{\left(3+3\right)}^{2}=930$$

$$F\left(\mathrm{EB}\right)=1\left(1+4\right)+6\left(1+9\right)+3\left(1+16\right)+3\left(4+4\right)+18\left(4+9\right)+1\left(4+16\right)+1\left(9+9\right)=502$$

### 2.3. Topological Descriptors of Dabrafenib

A kinase inhibitor called dabrafenib is used to treat people with some forms of thyroid cancer, non-small cell lung cancer, and melanoma. The same mutation was also applied to metastatic non-small cell lung cancer.

Using the data from the edge partition, we computed topological descriptors for the dabrafenib (DB) in this section. Let graph of DB with edge set *E*. Let $E_{m,n}$ are edges of $G_{2}$ with $\left|E_{1,3}\;\right|=4,\left|E_{1,4}\;\right|=5,\;\left|E_{2,2}\;\right|=6,\;\left|E_{2,3}\;\right|=13,\left|E_{2,4}\;\right|=1\;,\left|E_{3,3}\;\right|=7,\left|E_{3,4}\;\right|=2\;.\;\mathrm{By}\;\mathrm{applying}\;\mathrm{Definitions}\;1–8\;\mathrm{we}\;\mathrm{obtained}\;\mathrm{the}\;\mathrm{results}\;\mathrm{and}\;\mathrm{TDs}\;\mathrm{are}\;\mathrm{given}\;\mathrm{as}\;\mathrm{follows}:$

$$\mathrm{ABC}(\mathrm{DB})=4\sqrt{\frac{1+3-2}{1\times 3}\;}+5\sqrt{\frac{1+4-2}{1\times 4}\;}+6\sqrt{\frac{2+2-2}{2\times 2}\;}+13\sqrt{\frac{2+3-2}{2\times 3}\;}+1\sqrt{\frac{2+4-2}{2\times 4}\;}+7\sqrt{\frac{3+3-2}{3\times 3}}++2\sqrt{\frac{3+4-2}{3\times 4}\;}=27.70$$

$$RA\left(\mathrm{DB}\right)=4\sqrt{\frac{1}{1\times 3}\;}+5\sqrt{\frac{1}{1\times 4}\;}+6\sqrt{\frac{1}{2\times 2}\;}+13\sqrt{\frac{1}{2\times 3}}+1\sqrt{\frac{1}{2\times 4}\;}+7\sqrt{\frac{1}{3\times 3}\;}+2\sqrt{\frac{1}{3\times 4}\;}=16.38$$

$$S\left(\mathrm{DB}\right)=4\sqrt{\frac{1}{1+3}\;}+5\sqrt{\frac{1}{1+4}\;}+6\sqrt{\frac{1}{2+2}\;}+13\sqrt{\frac{1}{2+3}\;}+1\sqrt{\frac{1}{2+4}\;}+7\sqrt{\frac{1}{3+3}\;}+2\sqrt{\frac{1}{3+4}\;}=17.07$$

$$GA(\mathrm{DB})=\frac{4\sqrt{1\times 3}}{1+3}+\frac{10\sqrt{1\times 4}}{1+4}+\frac{12\sqrt{2\times 2}}{2+2}+\frac{26\sqrt{2\times 3}}{2+3}+\frac{2\sqrt{2\times 4}}{2+4}+\frac{14\sqrt{3\times 3}}{3+3}+\frac{4\sqrt{3\times 4}}{3+4}=36.12$$

$$M1\left(\mathrm{DB}\right)=4\left(1+3\right)+5\left(1+4\right)+6\left(2+2\right)+13\left(2+3\right)+1\left(2+4\right)+7\left(3+3\right)+2\left(3+4\right)=192$$

$$M2\left(\mathrm{DB}\right)=4\left(1\times 3\right)+5\left(1\times 4\right)+6\left(2\times 2\right)+13\left(2\times 3\right)+1\left(2\times 4\right)+7\left(3\times 3\right)+2\left(3\times 4\right)=229$$

$$H\left(\mathrm{DB}\right)=4\left(\frac{1}{1+3}\right)+5\left(\frac{1}{1+4}\right)+6\left(\frac{1}{2+2}\right)+13\left(\frac{1}{2+3}\right)+1\left(\frac{1}{2+4}\right)+7\left(\;\frac{1}{3+3}\right)+2\left(\frac{1}{3+4}\right)=15.44$$

$$HM\left(\mathrm{DB}\right)=4{\left(1+3\right)}^{2}+5\;{\left(1+4\right)}^{2}+6{\left(2+2\right)}^{2}+13{\left(2+3\right)}^{2}+1{\left(2+4\right)}^{2}+7{\left(3+3\right)}^{2}+2{\left(3+4\right)}^{2}=996$$

$$F\left(\mathrm{DB}\right)=4\left(1+9\right)+5\left(1+16\right)+6\left(4+4\right)+13\left(4+9\right)+1\left(4+16\right)+7\left(9+9\right)+2\left(9+16\right)=538$$

### 2.4. Topological Descriptors of Dacarbazine

Using the data from the edge partition, we computed topological descriptors for the dacarbazine (DZ) in this section. Let graph of DZ with edge set *E* and $E_{m,n}$ are edges in $G_{1}\;$with$,\;\left|E_{1,3}\;\right|=4,\;\left|E_{2,2}\;\right|=3,\;\left|E_{2,3}\;\right|=4,\left|E_{3,3}\;\right|=2$. By applying Definitions 1–8 we obtained the results and TDs are given as follows:

$$\mathrm{ABC}(\mathrm{DZ})=4\sqrt{\frac{1+3-2}{1\times 3}\;}+3\sqrt{\frac{2+2-2}{2\times 2}\;}+4\sqrt{\frac{2+3-2}{2\times 3}\;}+2\sqrt{\frac{3+3-2}{3\times 3}\;}=9.55$$

$$RA\left(\mathrm{DZ}\right)=4\sqrt{\frac{1}{1\times 3}\;}\;+3\sqrt{\frac{1}{2\times 2}\;}+4\sqrt{\frac{1}{2\times 3}}\;+2\sqrt{\frac{1}{3\times 3}\;}=6.11$$

$$S\left(\mathrm{DZ}\right)=\;4\sqrt{\frac{1}{1+3}\;}\;+3\sqrt{\frac{1}{2+2}\;}+4\sqrt{\frac{1}{2+3}\;}\;\;+2\sqrt{\frac{1}{3+3}\;}=6.11$$

$$GA\left(\mathrm{DZ}\right)=\;\;\frac{8\sqrt{1\times 3}}{1+3}+\frac{6\sqrt{2\times 2}}{2+2}+\frac{8\sqrt{2\times 3}}{2+3}+\frac{4\sqrt{3\times 3}}{3+3}=12.38$$

$$M1\left(\mathrm{DZ}\right)=4\left(1+3\right)+3\left(2+2\right)+4\left(2+3\right)+2\left(3+3\right)=60$$

$$M2\left(\mathrm{DZ}\right)=4\left(1\times 3\right)+3\left(2\times 2\right)+4\left(2\times 3\right)+2\left(3\times 3\right)=66$$

$$H\left(\mathrm{DZ}\right)=4\left(\frac{1}{1+3}\right)+3\left(\frac{1}{2+2}\right)+4\left(\frac{1}{2+3}\right)+2\left(\;\frac{1}{3+3}\right)=5.77$$

$$HM\left(\mathrm{DZ}\right)=4{\left(1+3\right)}^{2}+3{\left(2+2\right)}^{2}+4{\left(2+3\right)}^{2}+2{\left(3+3\right)}^{2}=284$$

$$F\left(\mathrm{DZ}\right)=4\left(1+9\right)+3\left(4+4\right)+4\left(4+9\right)+2\left(9+9\right)=152$$

### 2.5. Topological Descriptors of Fluorouracil

Using the data from the edge partition, we computed topological descriptors for the fluorouracil (FL) in this section. Let graph of FL with edge set *E*. Let $E_{m,n}$ are edges of $G_{2}$ with$\;\left|E_{1,3}\;\right|=3,\;\left|E_{2,2}\;\right|=1,\;\left|E_{2,3}\;\right|=4,\;\left|E_{3,3}\;\right|=$ 1. By applying Definitions 1–8 we obtained the results and TDs are given as follows:

$$\mathrm{ABC}(\mathrm{FL})=3\sqrt{\frac{1+3-2}{1\times 3}\;}+1\sqrt{\frac{2+2-2}{2\times 2}\;}+4\sqrt{\frac{2+3-2}{2\times 3}\;}+1\sqrt{\frac{3+3-2}{3\times 3}\;}=6.65$$

$$RA\left(\mathrm{FL}\right)=3\sqrt{\frac{1}{1\times 3}\;}\;+1\sqrt{\frac{1}{2\times 2}\;}+4\sqrt{\frac{1}{2\times 3}}\;+1\sqrt{\frac{1}{3\times 3}\;}=4.20$$

$$S\left(\mathrm{FL}\right)=\;3\sqrt{\frac{1}{1+3}\;}+1\sqrt{\frac{1}{2+2}\;}+4\sqrt{\frac{1}{2+3}\;}\;\;+1\sqrt{\frac{1}{3+3}\;}=4.20$$

$$\;GA\left(\mathrm{FL}\right)=\frac{6\sqrt{1\times 3}}{1+3}+\frac{2\sqrt{2\times 2}}{2+2}+\frac{4\sqrt{2\times 3}}{2+3}+\frac{2\sqrt{3\times 3}}{3+3}=8.52$$

$$M1\left(\mathrm{FL}\right)=3\left(1+3\right)+1\left(2+2\right)+4\left(2+3\right)+2\left(3+3\right)=42$$

$$\;M2\left(\mathrm{FL}\right)=3\left(1\times 3\right)+1\left(2\times 2\right)+4\left(2\times 3\right)+2\left(3\times 3\right)=46$$

$$H\left(\mathrm{FL}\right)=3\left(\frac{1}{1+3}\right)+1\left(\frac{1}{2+2}\right)+4\left(\frac{1}{2+3}\right)+2\left(\;\frac{1}{3+3}\right)=3.93$$

$$HM\left(\mathrm{FL}\right)=3{\left(1+3\right)}^{2}+1{\left(2+2\right)}^{2}+4{\left(2+3\right)}^{2}+2{\left(3+3\right)}^{2}=200$$

$$\;F\left(\mathrm{FL}\right)=3\left(1+9\right)++1\left(4+4\right)+4\left(4+9\right)++2\left(9+9\right)=108$$

### 2.6. Topological Descriptors of Trametinib

Using the data from the edge partition, we computed topological descriptors for the trametinib (TM) in this section. Let graph of TM with edge set *E* and $E_{m,n}$ are edges in $G_{1}\;$with$\;\left|E_{1,3}\;\right|=9,\left|E_{2,2}\;\right|=4,\;\left|E_{2,3}\;\right|=14,\;\left|E_{3,3}\;\right|=14$. By applying Definitions 1–8 we obtained the results and TDs are given as follows:

$$\mathrm{ABC}(\mathrm{TM})=9\sqrt{\frac{1+3-2}{1\times 3}\;}+4\sqrt{\frac{2+2-2}{2\times 2}\;}+14\sqrt{\frac{2+3-2}{2\times 3}\;}+14\sqrt{\frac{3+3-2}{3\times 3}\;}=29.41$$

$$RA\left(\mathrm{TM}\right)=9\sqrt{\frac{1}{1\times 3}\;}\;+4\sqrt{\frac{1}{2\times 2}\;}+14\sqrt{\frac{1}{2\times 3}}\;+14\sqrt{\frac{1}{3\times 3}\;}=17.58$$

$$S\left(\mathrm{TM}\right)=\;9\sqrt{\frac{1}{1+3}\;}\;+4\sqrt{\frac{1}{2+2}\;}+14\sqrt{\frac{1}{2+3}\;}\;\;+14\sqrt{\frac{1}{3+3}\;}=18.48$$

$$GA\left(\mathrm{TM}\right)=\frac{18\sqrt{1\times 3}}{1+3}+\frac{8\sqrt{2\times 2}}{2+2}+\frac{28\sqrt{2\times 3}}{2+3}+\frac{28\sqrt{3\times 3}}{3+3}=39.51$$

$$M1\left(\mathrm{TM}\right)=9\left(1+3\right)+4\left(2+2\right)+14\left(2+3\right)+14\left(3+3\right)=206$$

$$M2\left(\mathrm{TM}\right)=9\left(1\times 3\right)+4\left(2\times 2\right)+14\left(2\times 3\right)+14\left(3\times 3\right)=253$$

$$H\left(\mathrm{TM}\right)=9\left(\frac{1}{1+3}\right)+4\left(\frac{1}{2+2}\right)+14\left(\frac{1}{2+3}\right)+14\left(\;\frac{1}{3+3}\right)=16.77$$

$$HM\left(\mathrm{TM}\right)=9{\left(1+3\right)}^{2}+4{\left(2+2\right)}^{2}+14{\left(2+3\right)}^{2}+14{\left(3+3\right)}^{2}=1062$$

$$F\left(\mathrm{TM}\right)=9\left(1+9\right)+4\left(4+4\right)+14\left(4+9\right)+14\left(9+9\right)=556$$

### 2.7. Topological Descriptors of Daurismo

Using the data from the edge partition, we computed topological descriptors for the daurismo (DU) in this section. Let graph of DU with edge set *E*. Let $E_{m,n}$ are edges of $G_{2}$ with $\left|E_{1,3}\;\right|=3\;,$ $\left|E_{2,2}\;\right|=6,\;\left|E_{2,3}\;\right|=18,\;\left|E_{3,3}\;\right|=3$. By applying Definitions 1–8 we obtained the results and TDs are given as follows:

$$\mathrm{ABC}(\mathrm{DU})=3\sqrt{\frac{1+3-2}{1\times 3}\;}+6\sqrt{\frac{2+2-2}{2\times 2}\;}+18\sqrt{\frac{2+3-2}{2\times 3}\;}+3\sqrt{\frac{3+3-2}{3\times 3}\;}=21.42$$

$$RA\left(\mathrm{DU}\right)=3\sqrt{\frac{1}{1\times 3}\;}\;+6\sqrt{\frac{1}{2\times 2}\;}+18\sqrt{\frac{1}{2\times 3}}\;+3\sqrt{\frac{1}{3\times 3}\;}=13.08$$

$$S\left(\mathrm{DU}\right)=\;3\sqrt{\frac{1}{1+3}\;}\;+6\sqrt{\frac{1}{2+2}\;}+18\sqrt{\frac{1}{2+3}\;}\;\;+3\sqrt{\frac{1}{3+3}\;}=13.77$$

$$GA\left(\mathrm{DU}\right)=\;\;\frac{6\sqrt{1\times 3}}{1+3}+\frac{12\sqrt{2\times 2}}{2+2}+\frac{36\sqrt{2\times 3}}{2+3}+\frac{6\sqrt{3\times 3}}{3+3}=29.23$$

$$M1\left(\mathrm{DU}\right)=3\left(1+3\right)+6\left(2+2\right)+18\left(2+3\right)+3\left(3+3\right)=144$$

$$M2\left(\mathrm{DU}\right)=3\left(1\times 3\right)+6\left(2\times 2\right)+18\left(2\times 3\right)+3\left(3\times 3\right)=168$$

$$H\left(\mathrm{DU}\right)=3\left(\frac{1}{1+3}\right)+6\left(\frac{1}{2+2}\right)+18\left(\frac{1}{2+3}\right)+3\left(\;\frac{1}{3+3}\right)=12.70$$

$$HM\left(\mathrm{DU}\right)=3{\left(1+3\right)}^{2}+6{\left(2+2\right)}^{2}+18{\left(2+3\right)}^{2}+3{\left(3+3\right)}^{2}=702$$

$$F\left(\mathrm{DU}\right)=3\left(1+9\right)+6\left(4+4\right)+18\left(4+9\right)+3\left(9+9\right)=366$$

### 2.8. Topological Descriptors of Veurafenib

Using the data from the edge partition, we computed topological descriptors for the vemurafenib (VF) in this section. Let graph of VF with edge set *E*. Let $E_{m,n}$ are edges of $G_{2}$ with $\left|E_{1,2}\;\right|=1,\;\left|E_{1,3}\;\right|=4,\left|E_{1,4}\;\right|=2,\;\left|E_{2,2}\;\right|=6,\;\left|E_{2,3}\;\right|=13,\;\left|E_{2,4}\;\right|=2,\left|E_{3,3}\;\right|=8$. By applying Definitions 1–8 we obtained the results and TDs are given as follows:

$$\mathrm{ABC}(\mathrm{VF})=1\sqrt{\frac{1+2-2}{1\times 2}\;}+4\sqrt{\frac{1+3-2}{1\times 3}\;}+2\sqrt{\frac{1+4-2}{1\times 4}\;}+6\sqrt{\frac{2+2-2}{2\times 2}\;}+13\sqrt{\frac{2+3-2}{2\times 3}\;}+2\sqrt{\frac{2+4-2}{2\times 4}\;}+8\sqrt{\frac{3+3-2}{3\times 3}\;}=25.89$$

$$RA\left(\mathrm{VF}\right)=1\sqrt{\frac{1}{1\times 2}\;}+4\sqrt{\frac{1}{1\times 3}\;}\;+2\sqrt{\frac{1}{1\times 4}\;}+6\sqrt{\frac{1}{2\times 2}\;}+13\sqrt{\frac{1}{2\times 3}}+2\sqrt{\frac{1}{2\times 4}\;}\;+8\sqrt{\frac{1}{3\times 3}\;}=15.70$$

$$S\left(\mathrm{VF}\right)=\;1\sqrt{\frac{1}{1+2}\;}+4\sqrt{\frac{1}{1+3}\;}\;+2\sqrt{\frac{1}{1+4}\;}+6\sqrt{\frac{1}{2+2}\;}+13\sqrt{\frac{1}{2+3}\;}+2\sqrt{\frac{1}{2+4}\;}\;+8\sqrt{\frac{1}{3+3}\;}=16.37$$

$$GA\left(\mathrm{VF}\right)=\;\frac{2\sqrt{1\times 2}}{1+2}+\frac{8\sqrt{1\times 3}}{1+3}+\frac{4\sqrt{1\times 4}}{1+4}+\frac{12\sqrt{2\times 2}}{2+2}\;\frac{26\sqrt{2\times 3}}{2+3}+\frac{4\sqrt{2\times 4}}{2+4}+\frac{16\sqrt{3\times 3}}{3+3}=34.63$$

$$M1\left(\mathrm{VF}\right)=1\left(1+2\right)+4\left(1+3\right)+2\left(1+4\right)+6\left(2+2\right)+13\left(2+3\right)+2\left(2+4\right)+8\left(3+3\right)=178$$

$$M2\left(\mathrm{VF}\right)=1\left(1\times 2\right)+4\left(1\times 3\right)+2\left(1\times 4\right)+6\left(2\times 2\right)+13\left(2\times 3\right)+2\left(2\times 4\right)+8\left(3\times 3\right)=212$$

$$H\left(\mathrm{VF}\right)=1\left(\frac{1}{1+2}\right)+4\left(\frac{1}{1+3}\right)+2\left(\frac{1}{1+4}\right)+6\left(\frac{1}{2+2}\right)+13\left(\frac{1}{2+3}\right)+2\left(\frac{1}{2+4}\right)+8\left(\;\frac{1}{3+3}\right)=15.00$$

$$HM\left(\mathrm{VF}\right)=1{\left(1+2\right)}^{2}+4{\left(1+3\right)}^{2}+2\;{\left(1+4\right)}^{2}+6{\left(2+2\right)}^{2}+13{\left(2+3\right)}^{2}+2{\left(2+4\right)}^{2}+8{\left(3+3\right)}^{2}=904$$

$$\;F\left(\mathrm{VF}\right)=1\left(1+4\right)+4\left(1+9\right)+2\left(1+16\right)+6\left(4+4\right)+13\left(4+9\right)+2\left(4+16\right)+8\left(9+9\right)=480$$

### 2.9. Topological Descriptors of Imiquimod

Using the data from the edge partition, we computed topological descriptors for the imiquimod (IQ) in this section. Let graph of IQ with edge set *E* and $E_{m,n}$ are edges in $G_{1}\;$with $\left|E_{1,3}\;\right|=3,\left|E_{2,2}\;\right|=4,\;\left|E_{2,3}\;\right|=8,\;\left|E_{3,3}\;\right|=5$. By applying Definitions 1–8 we obtained the results and TDs are given as follows:

$$\mathrm{ABC}(\mathrm{IQ})=3\sqrt{\frac{1+3-2}{1\times 3}\;}+4\sqrt{\frac{2+2-2}{2\times 2}\;}+8\sqrt{\frac{2+3-2}{2\times 3}\;}+5\sqrt{\frac{3+3-2}{3\times 3}\;}=14.27$$

$$RA\left(\mathrm{IQ}\right)=3\sqrt{\frac{1}{1\times 3}\;}\;+4\sqrt{\frac{1}{2\times 2}\;}+8\sqrt{\frac{1}{2\times 3}}\;+5\sqrt{\frac{1}{3\times 3}\;}=8.66$$

$$S\left(\mathrm{IQ}\right)=\;3\sqrt{\frac{1}{1+3}\;}\;+4\sqrt{\frac{1}{2+2}\;}+8\sqrt{\frac{1}{2+3}\;}\;\;+5\sqrt{\frac{1}{3+3}\;}=9.12$$

$$GA\left(\mathrm{IQ}\right)=\;\frac{6\sqrt{1\times 3}}{1+3}+\frac{8\sqrt{2\times 2}}{2+2}+\frac{16\sqrt{2\times 3}}{2+3}+\frac{10\sqrt{3\times 3}}{3+3}=19.44$$

$$M1\left(\mathrm{IQ}\right)=3\left(1+3\right)+4\left(2+2\right)+8\left(2+3\right)+5\left(3+3\right)=98$$

$$M2\left(\mathrm{IQ}\right)=3\left(1\times 3\right)+4\left(2\times 2\right)+8\left(2\times 3\right)+5\left(3\times 3\right)=118$$

$$H\left(\mathrm{IQ}\right)=3\left(\frac{1}{1+3}\right)+4\left(\frac{1}{2+2}\right)+8\left(\frac{1}{2+3}\right)+5\left(\;\frac{1}{3+3}\right)=8.37$$

$$HM\left(\mathrm{IQ}\right)=3{\left(1+3\right)}^{2}+4{\left(2+2\right)}^{2}+8{\left(2+3\right)}^{2}+5{\left(3+3\right)}^{2}=492$$

$$F\left(\mathrm{IQ}\right)=3\left(1+9\right)+4\left(4+9\right)+8\left(4+16\right)+5\left(9+9\right)=256$$

### 2.10. Topological Descriptors of Odomzo

Using the data from the edge partition, we computed topological descriptors for the odomzo (OM) in this section. Let graph of OM with edge set *E*. Let $E_{m,n}$ are edges of $G_{2}$ with $\left|E_{1,3}\;\right|=4\;,$ $\left|E_{1,4}\;\right|=3,\;\left|E_{2,2}\;\right|=6,\;\left|E_{2,3}\;\right|=19,\left|E_{2,4}\;\right|=1,\left|E_{3,3}\;\right|=5$. By applying Definitions 1–8 we obtained the results and TDs are given as follows:

$$\mathrm{ABC}(\mathrm{OM})=4\sqrt{\frac{1+3-2}{1\times 3}\;}+3\sqrt{\frac{1+4-2}{1\times 4}\;}6\sqrt{\frac{2+2-2}{2\times 2}\;}+19\sqrt{\frac{2+3-2}{2\times 3}\;}+1\sqrt{\frac{2+4-2}{2\times 4}\;}+5\sqrt{\frac{3+3-2}{3\times 3}\;}=27.58$$

$$RA\left(\mathrm{OM}\right)=4\sqrt{\frac{1}{1\times 3}\;}\;+3\sqrt{\frac{1}{1\times 4}\;}+6\sqrt{\frac{1}{2\times 2}\;}+19\sqrt{\frac{1}{2\times 3}}+1\sqrt{\frac{1}{2\times 4}\;}\;+5\sqrt{\frac{1}{3\times 3}\;}=16.59$$

$$S\left(\mathrm{OM}\right)=\;4\sqrt{\frac{1}{1+3}\;}\;+3\sqrt{\frac{1}{1+4}\;}+6\sqrt{\frac{1}{2+2}\;}+19\sqrt{\frac{1}{2+3}\;}+1\sqrt{\frac{1}{2+4}\;}\;+5\sqrt{\frac{1}{3+3}\;}=17.29$$

$$GA\left(\mathrm{OM}\right)=\frac{8\sqrt{1\times 3}}{1+3}+\frac{6\sqrt{1\times 4}}{1+4}+\frac{12\sqrt{2\times 2}}{2+2}\;\frac{38\sqrt{2\times 3}}{2+3}+\frac{2\sqrt{2\times 4}}{2+4}+\frac{10\sqrt{3\times 3}}{3+3}=36.42$$

$$M1\left(\mathrm{OM}\right)=4\left(1+3\right)+3\left(1+4\right)+6\left(2+2\right)+19\left(2+3\right)+1\left(2+4\right)+5\left(3+3\right)=186$$

$$M2\left(\mathrm{OM}\right)=4\left(1\times 3\right)+3\left(1\times 4\right)+12\left(2\times 2\right)+19\left(2\times 3\right)+1\left(2\times 4\right)+5\left(3\times 3\right)=215$$

$$H\left(\mathrm{OM}\right)=4\left(\frac{1}{1+3}\right)+3\left(\frac{1}{1+4}\right)+6\left(\frac{1}{2+2}\right)+19\left(\frac{1}{2+3}\right)+1\left(\frac{1}{2+4}\right)+5\left(\;\frac{1}{3+3}\right)=15.80$$

$$HM\left(\mathrm{OM}\right)=4{\left(1+3\right)}^{2}+3\;{\left(1+4\right)}^{2}+6{\left(2+2\right)}^{2}+19{\left(2+3\right)}^{2}+1{\left(2+4\right)}^{2}+5{\left(3+3\right)}^{2}=926$$

$$F\left(\mathrm{OM}\right)=4\left(1+9\right)+3\left(1+16\right)+6\left(4+4\right)+19\left(4+9\right)+1\left(4+16\right)+5\left(9+9\right)=496$$

### 2.11. Topological Descriptors of Vismodegib

Using the data from the edge partition, we computed topological descriptors for the vismodegib (VD) in this section. Let graph of VD with edge set *E* and $E_{m,n}$ are edges in $G_{1}\;$with $\left|E_{1,3}\;\right|=3\;,\left|E_{1,4}\;\right|=5\;,\;\left|E_{2,2}\;\right|=6,\;\left|E_{2,3}\;\right|=12\;,\left|E_{3,3}\;\right|=4\;,\left|E_{3,4}\;\right|=1$. By applying Definitions 1–8 we obtained the results and TDs are given as follows:

$$\mathrm{ABC}(\mathrm{VD})=3\sqrt{\frac{1+3-2}{1\times 3}\;}+5\sqrt{\frac{1+4-2}{1\times 4}\;}+6\sqrt{\frac{2+2-2}{2\times 2}\;}+12\sqrt{\frac{2+3-2}{2\times 3}\;}+4\sqrt{\frac{3+3-2}{3\times 3}\;}+1\sqrt{\frac{3+4-2}{3\times 4}\;}=21.09$$

$$RA\left(\mathrm{VD}\right)=3\sqrt{\frac{1}{1\times 3}\;}\;+5\sqrt{\frac{1}{1\times 4}\;}+6\sqrt{\frac{1}{2\times 2}\;}+12\sqrt{\frac{1}{2\times 3}}\;+4\sqrt{\frac{1}{3\times 3}\;}+1\sqrt{\frac{1}{3\times 4}\;}=12.75$$

$$S\left(\mathrm{VD}\right)=\;3\sqrt{\frac{1}{1+3}\;}\;+5\sqrt{\frac{1}{1+4}\;}+6\sqrt{\frac{1}{2+2}\;}+12\sqrt{\frac{1}{2+3}\;}+4\sqrt{\frac{1}{2+4}\;}\;+1\sqrt{\frac{1}{3+3}\;}\;=13.22$$

$$GA\left(\mathrm{VD}\right)=\;\frac{6\sqrt{1\times 3}}{1+3}+\frac{10\sqrt{1\times 4}}{1+4}+\frac{12\sqrt{2\times 2}}{2+2}+\frac{24\sqrt{2\times 3}}{2+3}+\frac{16\sqrt{2\times 4}}{2+4}+\frac{2\sqrt{3\times 3}}{3+3}=27.75$$

$$M1\left(\mathrm{VD}\right)=3\left(1+3\right)+5\left(1+4\right)+6\left(2+2\right)+12\left(2+3\right)+4\left(2+4\right)+1\left(3+3\right)=142$$

$$M2\left(\mathrm{VD}\right)=3\left(1\times 3\right)+5\left(1\times 4\right)+6\left(2\times 2\right)+12\left(2\times 3\right)+4\left(2\times 4\right)+1\left(3\times 3\right)=165$$

$$H\left(\mathrm{VD}\right)=3\left(\frac{1}{1+3}\right)+5\left(\frac{1}{1+4}\right)+6\left(\frac{1}{2+2}\right)+12\left(\frac{1}{2+3}\right)+4\left(\frac{1}{2+4}\right)+1\left(\;\frac{1}{3+3}\right)=12.12$$

$$HM\left(\mathrm{VD}\right)=3{\left(1+3\right)}^{2}+5\;{\left(1+4\right)}^{2}+6{\left(2+2\right)}^{2}+12{\left(2+3\right)}^{2}+4{\left(2+4\right)}^{2}+1{\left(3+3\right)}^{2}=712$$

$$F\left(\mathrm{VD}\right)=3\left(1+9\right)+5\left(1+16\right)+6\left(4+4\right)+12\left(4+9\right)+4\left(4+16\right)+1\left(9+9\right)=382$$

### 2.12. Topological Descriptors of Cobimetinib

Using the data from the edge partition, we computed topological descriptors for the cobimetinib (CM) in this section. Let graph of VD with edge set *E* and $E_{m,n}$ are edges in $G_{1}\;$with $\left|E_{1,3}\;\right|=5\;,\left|E_{1,4}\;\right|=1\;,\;\left|E_{2,2}\;\right|=6,\;\left|E_{2,3}\;\right|=12,\;\left|E_{2,4}\;\right|=2,\left|E_{3,3}\;\right|=6\;,\left|E_{3,4}\;\right|=1$. By applying Definitions 1–8 we obtained the results and TDs are given as follows:

$$\mathrm{ABC}(\mathrm{CM})=5\sqrt{\frac{1+3-2}{1\times 3}\;}+1\sqrt{\frac{1+4-2}{1\times 4}\;}+6\sqrt{\frac{2+2-2}{2\times 2}\;}+12\sqrt{\frac{2+3-2}{2\times 3}\;}+2\sqrt{\frac{2+4-2}{2\times 4}\;}+6\sqrt{\frac{3+3-2}{3\times 3}\;}+1\sqrt{\frac{3+4-2}{3\times 4}\;}=23.00$$

$$RA\left(\mathrm{CM}\right)=5\sqrt{\frac{1}{1\times 3}\;}+1\sqrt{\frac{1}{1\times 4}\;}+6\sqrt{\frac{1}{2\times 2}\;}+12\sqrt{\frac{1}{2\times 3}}+2\sqrt{\frac{1}{2\times 4}\;}+6\sqrt{\frac{1}{3\times 3}\;}+1\sqrt{\frac{1}{3\times 4}\;}=14.28$$

$$S\left(\mathrm{CM}\right)=5\sqrt{\frac{1}{1+3}\;}+1\sqrt{\frac{1}{1+4}\;}+6\sqrt{\frac{1}{2+2}\;}+12\sqrt{\frac{1}{2+3}\;}+2\sqrt{\frac{1}{2+4}\;}+6\sqrt{\frac{1}{3+3}\;}+1\sqrt{\frac{1}{3+4}\;}=14.96$$

$$GA\left(\mathrm{CM}\right)=\frac{10\sqrt{1\times 3}}{1+3}+\frac{4\sqrt{1\times 4}}{1+4}+\frac{12\sqrt{2\times 2}}{2+2}+\frac{24\sqrt{2\times 3}}{2+3}+\frac{4\sqrt{2\times 4}}{2+4}+\frac{12\sqrt{3\times 3}}{3+3}+\frac{2\sqrt{3\times 4}}{3+4}=31.76$$

$$M1\left(\mathrm{CM}\right)=5\left(1+3\right)+1\left(1+4\right)+6\left(2+2\right)+12\left(2+3\right)+4\left(2+4\right)+12\left(3+3\right)+1\left(3+4\right)=164$$

$$M2\left(\mathrm{CM}\right)=5\left(1\times 3\right)+1\left(1\times 4\right)+12\left(2\times 2\right)+12\left(2\times 3\right)+2\left(2\times 4\right)+6\left(3\times 3\right)+1\left(3\times 4\right)=197$$

$$H\left(\mathrm{CM}\right)=5\left(\frac{1}{1+3}\right)+1\left(\frac{1}{1+4}\right)+6\left(\frac{1}{2+2}\right)+12\left(\frac{1}{2+3}\right)+2\left(\frac{1}{2+4}\right)+6\left(\;\frac{1}{3+3}\right)+1\left(\frac{1}{3+4}\right)=13.65$$

$$HM\left(\mathrm{CM}\right)=5{\left(1+3\right)}^{2}+1\;{\left(1+4\right)}^{2}+6{\left(2+2\right)}^{2}+12{\left(2+3\right)}^{2}++2{\left(2+4\right)}^{2}+6{\left(3+3\right)}^{2}+1{\left(3+4\right)}^{2}=838$$

$$F\left(\mathrm{CM}\right)=5\left(1+9\right)+1\left(1+16\right)++6\left(4+4\right)+12\left(4+9\right)+2\left(4+16\right)+6\left(9+9\right)+1\left(9+16\right)=444$$

### 2.13. Topological Descriptors of Vemurafenib

Using the data from the edge partition, we computed topological descriptors for the vemurafenib (VD) in this section. Let graph of VD with edge set *E* and $E_{m,n}$ are edges in $G_{1}\;$with, $\left|E_{1,2}\;\right|=1,\;\left|E_{1,3}\;\right|=4\;,$ $\left|E_{1,4}\;\right|=2,\;\left|E_{2,2}\;\right|=6,\;\left|E_{2,3}\;\right|=13,\;\left|E_{2,4}\;\right|=2,\;\left|E_{3,3}\;\right|=8\;.$By applying Definitions 1–8 we obtained the results and TDs are given as follows:

$$\mathrm{ABC}(G_{1})=1\sqrt{\frac{1+2-2}{1\times 2}\;}+4\sqrt{\frac{1+3-2}{1\times 3}\;}+2\sqrt{\frac{1+4-2}{1\times 4}\;}+6\sqrt{\frac{2+2-2}{2\times 2}\;}+13\sqrt{\frac{2+3-2}{2\times 3}\;}+2\sqrt{\frac{2+4-2}{2\times 4}\;}+8\sqrt{\frac{3+3-2}{3\times 3}\;}=25.89$$

$$RA\left(GG_{1}\right)=1\sqrt{\frac{1}{1\times 2}\;}+4\sqrt{\frac{1}{1\times 3}\;}\;+2\sqrt{\frac{1}{1\times 4}\;}+6\sqrt{\frac{1}{2\times 2}\;}+13\sqrt{\frac{1}{2\times 3}}+2\sqrt{\frac{1}{2\times 4}\;}\;+8\sqrt{\frac{1}{3\times 3}\;}=15.70$$

$$S\left(G_{1}\right)=\;1\sqrt{\frac{1}{1+2}\;}+4\sqrt{\frac{1}{1+3}\;}\;+2\sqrt{\frac{1}{1+4}\;}+6\sqrt{\frac{1}{2+2}\;}+13\sqrt{\frac{1}{2+3}\;}+2\sqrt{\frac{1}{2+4}\;}\;+8\sqrt{\frac{1}{3+3}\;}=16.37$$

$$GA\left(G_{1}\right)=\;\frac{2\sqrt{1\times 2}}{1+2}+\frac{8\sqrt{1\times 3}}{1+3}+\frac{4\sqrt{1\times 4}}{1+4}+\frac{12\sqrt{2\times 2}}{2+2}+\frac{26\sqrt{2\times 3}}{2+3}+\frac{4\sqrt{2\times 4}}{2+4}+\frac{16\sqrt{3\times 3}}{3+3}=34.63$$

$$M1\left(G_{1}\right)=1\left(1+2\right)+4\left(1+3\right)+2\left(1+4\right)+6\left(2+2\right)+13\left(2+3\right)+2\left(2+4\right)+8\left(3+3\right)=178$$

$$M2\left(G_{1}\right)=1\left(1\times 2\right)+4\left(1\times 3\right)+2\left(1\times 4\right)+6\left(2\times 2\right)+13\left(2\times 3\right)+2\left(2\times 4\right)+8\left(3\times 3\right)=212$$

$$H\left(G_{1}\right)=1\left(\frac{1}{1+2}\right)+4\left(\frac{1}{1+3}\right)+2\left(\frac{1}{1+4}\right)+6\left(\frac{1}{2+2}\right)+13\left(\frac{1}{2+3}\right)+2\left(\frac{1}{2+4}\right)+8\left(\;\frac{1}{3+3}\right)=15.00$$

$$HM\left(G_{1}\right)=1{\left(1+2\right)}^{2}+4{\left(1+3\right)}^{2}+2\;{\left(1+4\right)}^{2}+6{\left(2+2\right)}^{2}+13{\left(2+3\right)}^{2}+2{\left(2+4\right)}^{2}+8{\left(3+3\right)}^{2}=904$$

$$F\left(G_{1}\right)=1\left(1+4\right)+4\left(1+9\right)+2\left(1+16\right)+6\left(4+4\right)+13\left(4+9\right)+2\left(4+16\right)+8\left(9+9\right)=480$$

### 2.14. Topological Descriptors of Picatio

Using the data from the edge partition, we computed topological descriptors for the picato (PC) in this section. Let $G_{1}$ be a graph of PC with edge set *E* and $E_{m,n}$ are edges in $G_{1}\;$ with, $\left|E_{1,2}\;\right|=2\;,\;\left|E_{1,3}\;\right|=6\left|E_{1,4}\;\right|=3,\;\left|E_{2,3}\;\right|=9,\left|E_{2,4}\;\right|=1,\left|E_{3,3}\;\right|=7,\left|E_{3,4}\;\right|=5\;,\left|E_{4,4}\;\right|=1$. By applying Definitions 1–8 we obtained the results and TDs are given as follows:

$$\mathrm{ABC}(\mathrm{PC})=2\sqrt{\frac{1+2-2}{1\times 2}\;}+6\sqrt{\frac{1+3-2}{1\times 3}\;}+3\sqrt{\frac{1+4-2}{1\times 4}\;}+9\sqrt{\frac{2+3-2}{2\times 3}\;}+1\sqrt{\frac{2+4-2}{2\times 4}\;}+7\sqrt{\frac{3+3-2}{3\times 3}\;}+5\sqrt{\frac{3+4-2}{3\times 4}\;}+1\sqrt{\frac{4+4-2}{4\times 4}\;}=24.49$$

$$RA\left(\mathrm{PC}\right)=2\sqrt{\frac{1}{1\times 2}\;}+6\sqrt{\frac{1}{1\times 3}\;}\;+3\sqrt{\frac{1}{1\times 4}\;}+9\sqrt{\frac{1}{2\times 3}}+1\sqrt{\frac{1}{2\times 4}\;}\;+7\sqrt{\frac{1}{3\times 3}\;}+5\sqrt{\frac{1}{3\times 4}\;}+1\sqrt{\frac{1}{4\times 4}\;\;}=14.43$$

$$S\left(\mathrm{PC}\right)=2\;\sqrt{\frac{1}{1+2}\;}+6\sqrt{\frac{1}{1+3}\;}\;+3\sqrt{\frac{1}{1+4}\;}+9\sqrt{\frac{1}{2+3}\;}+1\sqrt{\frac{1}{2+4}\;}\;+7\sqrt{\frac{1}{3+3}\;}+5\sqrt{\frac{1}{3+4}\;}+1\sqrt{\frac{1}{4+4}\;\;}=15.03$$

$$GA\left(\mathrm{PC}\right)=\;\frac{4\sqrt{1\times 2}}{1+2}+\frac{12\sqrt{1\times 3}}{1+3}+\frac{6\sqrt{1\times 4}}{1+4}+\frac{18\sqrt{2\times 3}}{2+3}+\frac{2\sqrt{2\times 4}}{2+4}+\frac{14\sqrt{3\times 3}}{3+3}+\frac{10\sqrt{3\times 4}}{3+4}+\frac{2\sqrt{4\times 4}}{4+4}=32.19$$

$$M1\left(\mathrm{PC}\right)=2\left(1+2\right)+6\left(1+3\right)+3\left(1+4\right)+9\left(2+3\right)+1\left(2+4\right)+7\left(3+3\right)+5\left(3+4\right)+1\left(4+4\right)=181$$

$$M2\left(\mathrm{PC}\right)=2\left(1\times 2\right)+6\left(1\times 3\right)+3\left(1\times 4\right)+9\left(2\times 3\right)+1\left(2\times 4\right)+7\left(3\times 3\right)+5\left(3\times 4\right)+1\left(4\times 4\right)=235$$

$$H\left(\mathrm{PC}\right)=2\left(\frac{1}{1+2}\right)+6\left(\frac{1}{1+3}\right)+3\left(\frac{1}{1+4}\right)+9\left(\frac{1}{2+3}\right)+1\left(\frac{1}{2+4}\right)+7\left(\;\frac{1}{3+3}\right)+5\left(\frac{1}{3+4}\right)+1\left(\frac{1}{4+4}\right)=13.48$$

$$F\left(\mathrm{PC}\right)=2\left(1+4\right)+5\left(1+9\right)+3\left(1+16\right)+11\left(4+9\right)+1\left(4+16\right)+5\left(9+9\right)+5\left(9+16\right)+1\left(16+16\right)=541$$

## 3. Quantitative Structure Analysis and Regression Models

We have used some regression models between calculated topological descriptors and physicochemical attributes derived from PubChem in order to determine the utility of a topological index. Calculations of the aforementioned TDs and the physicochemical characteristics of molecular structures have been tabulated accordingly in Table 1.

**Figure 2:**
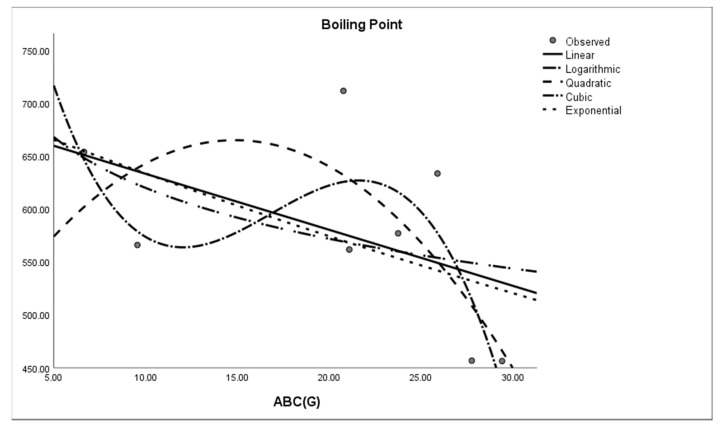
Exponential regression model of ABC (G) with boiling point.

**Figure 3:**
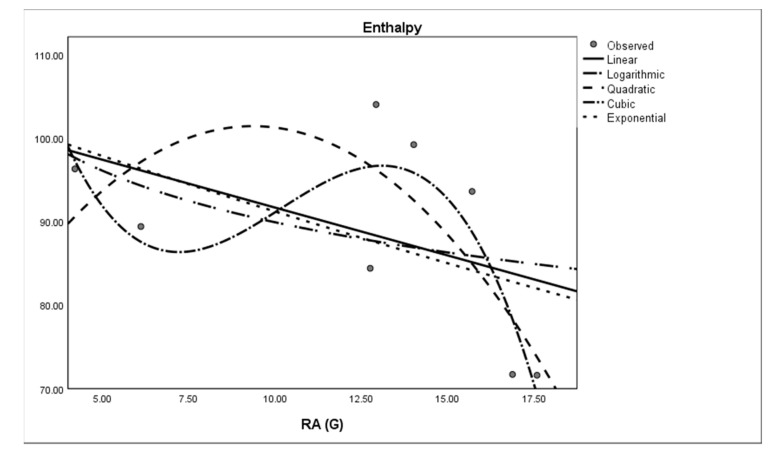
Quadratic regression model of ABC (G) with enthalpy.

**Figure 4:**
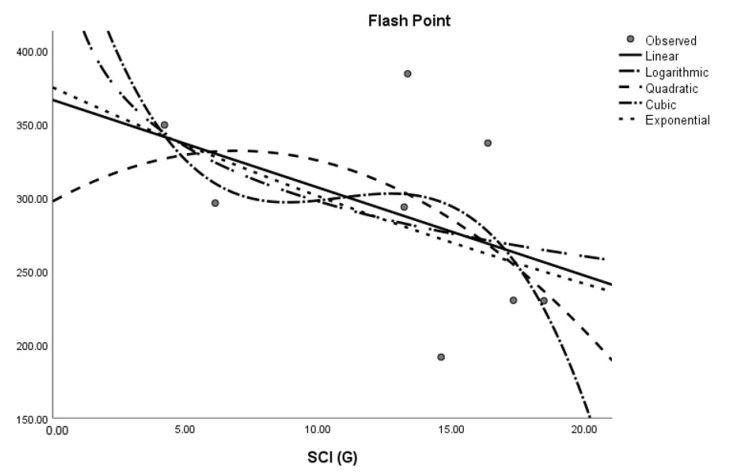
Logarithmic regression model of ABC (G) with flash point.

**Figure 5:**
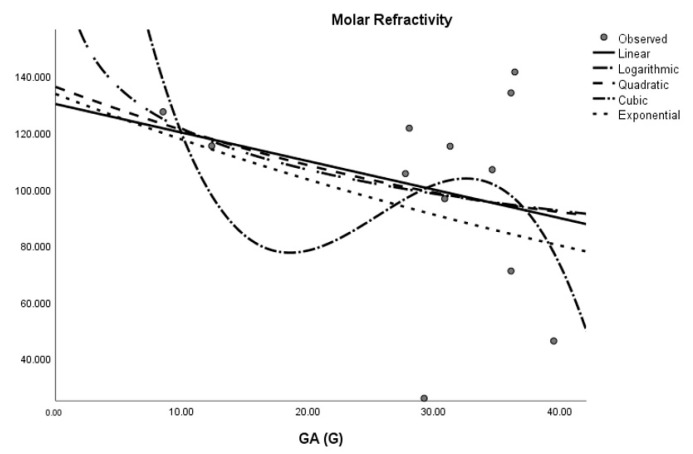
Cubic regression model of ABC (G) with refractivity.

**Figure 6:**
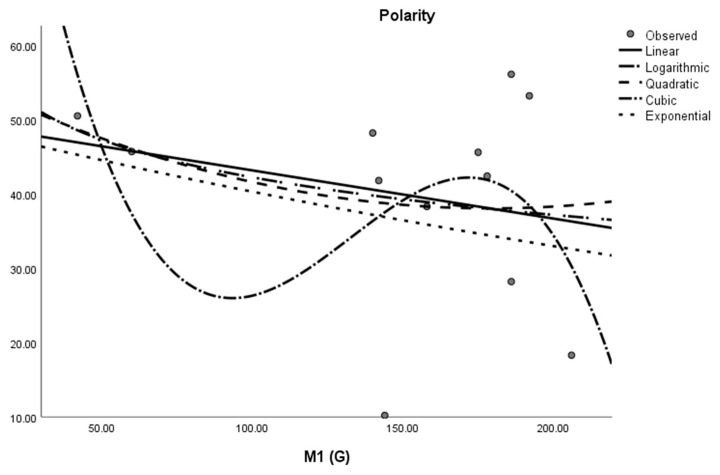
Cubic regression model of ABC (G) with polarity.

**Figure 7:**
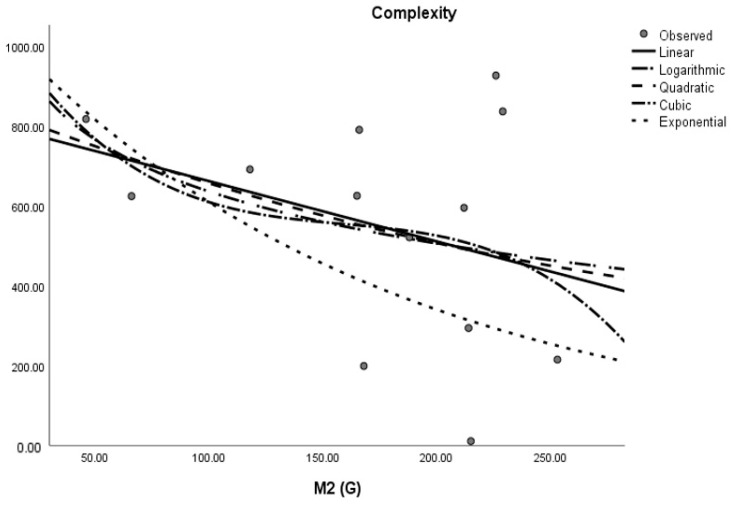
Quadratic regression model of ABC (G) with complexity.

Regression models are used to fit the curves, thus, we looked into the exponential, logarithmic, cubic, quadratic, and linear models. Here, we have highlighted a few top topological index regression model predictors for this specific physicochemical feature. As a result, the regression model is the best to test and use for this analysis. We used some regression models to fit curves rather than straight lines. We tested the following equations:

$$M=a+c_{1}\;N_{1}\;(\mathrm{Linear}\;\mathrm{Equation})$$

$$M=a+c_{1}\;N_{1}+c_{2}\;N_{2}\;(\mathrm{Quadratic}\;\mathrm{Equation})$$

$$M=a+c_{1}\;N_{1}+c_{2}\;N_{2}+c_{3}\;N_{3}\;\;(\mathrm{Cubic}\;\mathrm{Equation})$$

where *M* is the dependent variable, a is the regression model constant, *N_i_* (i = 1, 2, 3. . . ) are independent variables, *c_i_* (i = 1, 2, 3. . . ) are the coefficients for the individual descriptor is the number of samples used for building the regression equation. The curvilinear regression analyses and other results were obtained by using SPSS statistical software and graphical representation is presented in Figure 2, Figure 3, Figure 4, Figure 5, Figure 6 and Figure 7. The curvilinear regression models’ independent variables are the Randic index, the first and second Zagreb indices, the GA index, the ABC index, the Forgotten index, and the Harmonic index of thirteen skin cancer drugs.

## 4. Graphical Comparison

**Figure 8:**
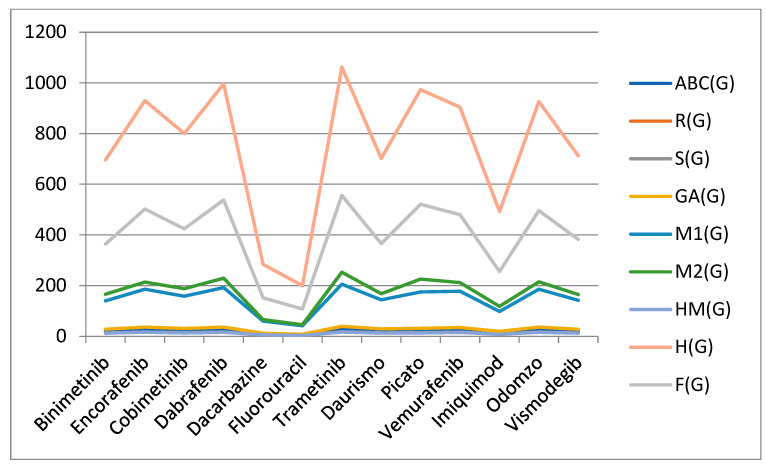
Graph of medicines with TDs.

We have supplied data for topological indices for the structure of skin cancer medications in order to comprehend the parallels between the biological and statistical behavior of the two chemical substances and graphical representation is presented in Figure 8.

## 5. Conclusions

To further understand how the biological and statistical behavior of the two chemical substances are similar, we developed topological descriptors for skin cancer in this article. The topological descriptors’ graphical behavior when used to forecast physical, chemical, and biological qualities was computed above. The QSPR study has shown that molecular descriptors (TDs) are the best tools to predict the physicochemical properties of drugs used for medical and pharmaceutical characteristics. Boiling point, molar refractivity, and complexity are better reflected whereas other polarities and polar surface areas are not estimated. In a quadratic regression model, molecular descriptor S (G) is best predicted with refractivity, melting point, and complexity. In a logarithmic regression model, molecular descriptor ABC (G) is best predicted with refractivity. In an exponential regression model, molecular descriptors M1 (G) and HM (G) are best predicted with molar refractivity. The pharmaceutical sector will be able to produce fresh treatments that will undoubtedly be beneficial in acquiring preventive measures for the aforementioned sickness with the calculated value derived from this. They provide techniques for estimating attributes for fresh exposures to various diseases. It will be useful in determining and forecasting a variety of properties and processes, such as entropy, critical pressure, boiling point, acentric factor, enthalpy, and others. Our discoveries may also help in the development of new medications for the treatment of skin cancer drugs.

## Figures and Tables

**Table 1:** Physical properties of drugs.

Name of Drug	Boiling Point (°C)	$\mathrm{Molar}\;\mathrm{Refractivity}\;(cm^{3})$	Polarity $\left(cm^{3}\right)$	Complexity	$\mathrm{Polar}\;\mathrm{Surface}\;\mathrm{Area}\;(A^{2})$
Binimetinib	711.4	121.6	48.2	790	100
Encorafenib	456.7	71	28.2	294	57
Cobimetinib		96.6	38.3	521	88
Dabrafenib		134.1	53.2	836	149
Dacarbazine	565.9	115.3	45.7	624	65
Fluorouracil	653.7	127.4	50.5	817	147
Trametinib	456.3	46.2	18.3	215	100
Daurismo		25.9	10.2	199	58
Picato	576.9	115.2	45.6	926	104
Vemurafenib	633.4	106.9	42.4	595	97
Imiquimod				691	
Odomzo		141.5	56.1	10.9	102
Vismodegib	561.6	105.5	41.8	625	85

## Data Availability

All data is available in the manuscript.

## Sample Availability

Samples of the compounds are not available from the authors.
